# Possible Therapeutic Options for Complex Regional Pain Syndrome

**DOI:** 10.3390/biomedicines9060596

**Published:** 2021-05-24

**Authors:** Myeounghoon Cha, Kyung Hee Lee, Minjee Kwon, Bae Hwan Lee

**Affiliations:** 1 Department of Physiology, College of Medicine, Yonsei University, Seoul 03722, Korea; 2 Department of Dental Hygiene, Division of Health Science, Dongseo University, Busan 47011, Korea; kyhee@gdsu.dongseo.ac.kr; 3 Department of Nursing, Kyungil University, Gyeongsan 38428, Korea; mjkwon@kiu.kr; 4 Brain Korea 21 PLUS Project for Medical Science, College of Medicine, Yonsei University, Seoul 03722, Korea

**Keywords:** complex regional pain syndrome, chronic post-ischemic pain, URB597, pyrrolidine dithiocarbamate, hydralazine

## Abstract

Complex regional pain syndrome (CRPS) describes an array of painful conditions that are characterized by continuing regional pain. CRPS comprises severe and inappropriate pain in cases of complete recovery after trauma. Research on the pharmacological treatment of CRPS, however, has not been well investigated. In this study, we compared the pain relief effects of different drugs (URB597, pyrrolidine dithiocarbamate, and hydralazine) in a rat model of chronic post-ischemic pain-induced CRPS. After drug injection, CRPS-induced mechanical allodynia was significantly recovered. After three repetitive drug injections, mechanical sensitivity generally improved as hyper-nociception subsided. Reduced Nav1.7 expression at dorsal root ganglions (DRGs) was observed in the drug treatment groups. Neural imaging analysis revealed decreased neural activity for each drug treatment, compared to vehicle. In addition, treatments significantly reduced IL-1β, IL-6, and TNFα expression in DRGs. These results indicated that drugs could reduce the expression of inflammatory factors and alleviate the symptoms of chronic post-ischemic pain-induced CRPS.

## 1. Introduction

Complex regional pain syndrome (CRPS) is a type of a neuropathic pain disease also called reflex sympathetic dystrophy or causalgia [1]. CRPS is a chronic pain disease that typically occurs after a stroke, spinal cord injury, or myocardial infarction. CRPS is largely classified into CRPS type I (CRPS1, reflex sympathetic dystrophy) and CRPS type II (CRPS2, causalgia) [1,2]. Compared to CRPS2, which is caused by direct nerve damage, CRPS1 involves relatively minor trauma or fall accidents and complex symptoms arising after surgery. CRPS is defined as unidentified persistent pain and reportedly present as continuous and serious pain accompanied by abnormalities in autonomic nervous system and normal movement [3]. CRPS patients account for 26.2 per 100,000 people in the United States. Unfortunately, however, CRPS remains a refractory diseases for which there is no effective treatment method [4].

For clinical treatment of CRPS1, attempts to reduce pain by injecting anesthetic drugs, such as ketamine or dexmedetomidine, have been made [5,6]; however, the effect thereof appears to be limited, with some cases showing no therapeutic effect at all. Cases of failure have also been reported [6]. Thus, it can be assumed that CRPS1 is not a disease arising from a simple cause and that it is a symptom of two or more complex mechanisms. As such, various studies to observe the histological changes that appear at the onset of CRPS1 and the perception of pain in the brain have been conducted to investigate mechanisms of pain in and treatment methods for CRPS [7,8,9]. Recently, new studies have reported that deep-tissue microvascular disfunctions in muscles and tissues could induce CRPS1 [10,11]. In these studies, acute inflammation and edema due to tissue damage caused ischemia-reperfusion injury (I-R injury) through abnormal pathways and changes in the nervous system, including bones and muscles. The authors indicated that this could develop into CRPS1 by inducing microvascular changes.

Existing CRPS1 pain treatment involves medication, physical therapy, and psychological therapy, and if the pain is not relieved, therapeutic neuroablation or neurostimulation is performed or analgesics are administered using drug pumps [12]. In a clinical study of pain-reducing effects in CRPS1, the administration of a combination of gabapentin, tramadol, baclofen, and mexiletine was found to be of use when pain is severe [13,14,15]. However, most of these drugs, including gabapentin, exhibit withdrawal symptoms, such that administration of them is limited. Recently, clinical results have also reported reduced pain with injections of ketamine, a selective NMDA antagonist in CRPS1, although some patients with CRPS1 have reported no effect [16,17].

These therapeutic difficulties are due to insufficient evidence on the pathogenesis and characteristics of CRPS1. Therefore, in this study, we aimed to investigate the analgesic effects of CRPS1 in depth using a rat model, observing pain behaviors, physiological and histological changes, and alteration of inflammatory factors in dorsal root ganglions (DRGs) in an attempt to identify potentially effective therapeutics for treating CRPS1. Here, we noted pain relief effects in CRPS1 patients with the use of URB597 to control fatty acid amide hydrolase (FAAH) in the endocannabinoid system. In addition, in order to alleviate the inflammatory symptoms that may appear due to I-R injury, pyrrolidine dithiocarbamate (PDTC), an inhibitor of NF-kB, which is a mediator of the inflammatory response, was injected to reduce inflammatory responses in tissues to obtain a pain relief effect. Finally, as a method of further pain treatment, vasodilator hydralazine was administered to alleviate symptoms stemming from microvascular I-R injury, which appears to be the cause of CRPS1, through local vasodilation effects.

## 2. Materials and Methods

### 2.1. Animals and CRPS Model

Male SD rats (280–300 g, Koatec, Pyeongtaek, Korea) arrived 7 days before the start of experiments. They were kept under a 12/12 h light-dark cycle (lights on at 8:00 h) with free access to food and water. Chronic post-ischemia pain (CPIP) was induced by I-R injury to the left hind paw as described previously [18,19]. Briefly, animals were anesthetized with sodium pentobarbital (50 mg/kg, i.p.). After induction of anesthesia, a nitrile butadiene rubber 70 O-ring (SHEMEKS, Hwaseong, Korea) with an internal diameter of 4.8 mm was placed around the rat’s left ankle joint. After 3 h, the O-ring was cut, allowing reperfusion of the hind limb [18].

### 2.2. Drug Administration and Mechanical Allodynia Assessment

Mechanical allodynia was assessed by measuring mechanical withdrawal threshold values using an electronic von Frey filament (no.38450; UGO Basile, Varese, Italy). Rats were individually placed in an acrylic cage and habituated for 10 minutes. The filament was vertically applied to the planta skin of the left hind paw, and force values were measured until the animal exhibited withdrawal or licking of the hind paw. The measurements were performed seven times in 2- to 3-min intervals, and averaged data were collected, omitting the maximum and minimum values. All of the von Frey tests were assessed by a researcher who was blind to the experimental groups. For drug injections, URB597 (0.3 mg/kg, Cayman Chemical, Ann Arbor, MI, US) and PDTC (pyrrolidine dithiocarbamate, 200 mg/kg, Abcam, Cambridge, UK) were administered intraperitoneally daily for 3 days after the induction of CPIP. Additionally, hydralazine was administered at 2 mL/kg for 3 days, which constituted a dose of 25 mg/kg of hydralazine. Nocifensive behavior was tested before drug injection and at 5 days post initial injection. During the 5 days, each drug was injected on days 0–2.

### 2.3. Immunohistochemistry

Immunohistochemistry was conducted to verify inflammatory responses in L4 and L5 DRGs. Under urethane anesthesia (1.25 g/kg, i.p.), each rat was transcardially perfused with normal saline (0.9% NaCl), followed by 4% paraformaldehyde in 0.1 M sodium phosphate buffer (PB, pH 7.4). DRGs were removed and immersed in 4% paraformaldehyde in 0.1 M PB for 24 h at 4 °C for post-fixation. The tissues were kept in 30% sucrose in phosphate buffer (PB, pH 7.4) at 4 °C. For immunostaining, embedded tissues were cryosectioned to 10 μm (HM 525, Thermo Scientific, Waltham, MA, US). The section slides were incubated overnight at 4 °C with primary antibodies against Nav1.7 (1:500; cell signaling technology, Danvers, MA, USA), washed with PBS, and incubated for 2 h at room temperature with Alexa Fluor 488 secondary antibodies (1:1000; Jackson ImmunoResearch, West Grove, PA, USA). DAPI was used for counterstaining. Immunofluorescent sections were imaged by LSM700 confocal microscope (Zeiss, Oberkochen, Germany) using 10× and 40× PlanApo oil-immersion lenses. Briefly, 12 μm-thick confocal Z-stacks of the synaptic zone in ZI were captured. Three image stacks per rat (4/group) were used for analysis, and the number of cells with Nav1.7 were quantified.

### 2.4. Voltage-Sensitive Dye Imaging

Voltage-sensitive dye (VSD) imaging was performed as described in our previous report [20]. Rats were fully anesthetized with urethane (1.25 g/kg, i.p.), after which the lumbar spinal cord and L4–L5 DRGs (identified by tracing the spinal roots back to the sciatic nerve) were dissected. Therefrom, blood and connective tissue were removed in 4 °C saline. The DRG tissue was then stored at room temperature for 1 hour. DRG was stained using a voltage-sensitive dye (di-2-ANEPEQ, 50 mg/mL in saline; Molecular Probes, Eugene, OR, US) for 1 hour and carefully rinsed with normal saline. Optical imaging was performed directly on the exposed DRGs with the application of single electrical stimulation (200-ms delay, 3-ms width, 3-mA intensity, and 3-s interstimulus interval). Dye fluorescence was detected using a high-resolution charge-coupled device camera (Brainvision Inc., Tokyo, Japan) equipped with a dichroic mirror with a 510–550 nm excitation filter and a 590 nm absorption filter. A tungsten halogen lamp (150 W) was used for fluorescence excitation. Optical signals were acquired at a rate of 3.7 ms/frame and averaged 15 times by a recording system (MiCAM02; Brainvision Inc.). The amplitudes and excitatory areas of optical signals were measured using a spatial filter (9  ×  9 pixels), and changes in optical intensity in the DRG were calculated as the percentage of fractional changes in fluorescence intensity (ΔF/F  ×  100). Data were analyzed using BV Analyzer software (Brainvision Inc., Tokyo, Japan).

### 2.5. Western Blot

To analyze inflammatory factors changes, samples were collected from the ipsilateral DRGs after CPIP. The L3, L4 and L5 DRGs of CPIP rats were collected and stored at −80 °C in polypropylene tubes until ready for Western blot analysis. The microtubes were centrifuged for 5 min at 10,000× *g*, and samples were collected. The samples were denatured in lithium dodecyl sulfate buffer containing DTT and loaded onto a NuPAGE® 4–12% Bis-Tris Mini gel to perform electrophoresis. Proteins were then transferred to polyvinylidene difluoride (PVDF) membranes (0.45 μm, Merck KGaA, Darmstadt, Germany). Membranes were blocked with 5% BSA in TBST and incubated overnight at 4 °C with a rabbit polyclonal primary antibody (IL-1β; Abcam ab9722, IL-6; Abcam 208113, TNFα; Abcam 66579, Cambridge, UK). Next, the membranes were washed and incubated with an anti-rabbit IgG HRP-conjugated secondary antibody for 1 h at room temperature. Immuno-reactive proteins were revealed by enhanced chemiluminescence. Bands recognized by the primary antibody were visualized using LAS-4000 (Fuji Film Co, Ltd., Tokyo, Japan), and densitometry was measured with Multi Gauge software (Fuji Film Co, Ltd., Tokyo, Japan). To allow quantification across several gels, one sample was used as an internal calibrator and was loaded on each gel and set to 100%.

### 2.6. Statistical Analysis

The results of the mechanical threshold test, VSD imaging analysis, and Western blotting for each experimental group are expressed as the mean and standard error of the mean (SEM). Analysis of variance (ANOVA) with a Tukey post hoc test was used to quantify differences in behavioral tests, immunohistochemistry results, optical imaging, and Western blot analysis. Statistical analyses were performed using GraphPad Prism version 8.0 (GraphPad Software, San Diego, CA, US). All *p*-values ≤ 0.05 were considered statistically significant.

## 3. Results

### 3.1. Establishment of the CPIP Model and Nocifensive Behavior Changes

**Figure 1:**
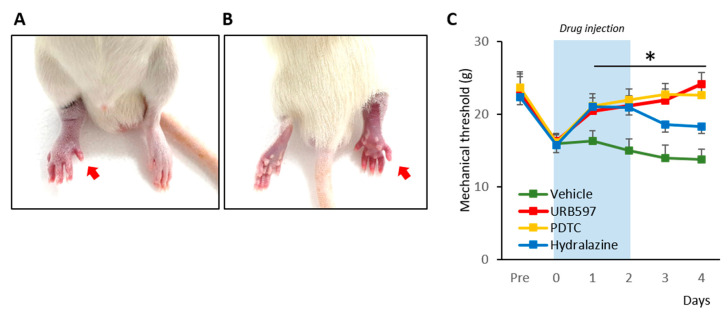
Rat model of chronic post-ischemia pain (CPIP) and nocifensive behavior changes. (**A**). Representative photographs of the rat hind paw taken during CPIP model establishment. The red arrow indicates the paw treated with an O-ring. (**B**). Hind-view of the paw with the O-ring. (**C**). Mechanical threshold changes pre- and post-CPIP. *n* = 6 rats/group; one-way ANOVA followed by Tukey post hoc test was used for statistical analysis; * *p* < 0.05.

To begin, we established a rat model of CRPS according to methods described before [18,21]. The hind paw of the CPIP rats showed clear evidence of hypoxia, becoming cold and cyanotic when the O-ring was placed (Figure 1A,B). After reperfusion, there was a period of hyperemia, and vasodilatation was observed. The edema persisted for 3 days and gradually returned back to normal. These observations are consistent with previous results and indicated the establishment of CRPS in the rats [18].

We proceeded to examine the effects of drugs (hydralazine, PDTC, and URB597) on the mechanical allodynia of CRPS rats. The nocifensive behavior changes from pre- to post-drug injection were compared for 6 consecutive days (Figure 1C). Pre-injection, randomly divided groups of rats showed similar mechanical threshold values (Pre-vehicle: 22.27 ± 2.33; Pre-URB597: 22.87 ± 2.32; Pre-PDTC: 23.65 ± 2.17; Pre-hydralazine: 22.37 ± 2.52). However, at 3 h after the induction of CPIP, each rat showed edema with reduced mechanical threshold (0 vehicle: 16.00 ± 1.20; 0 URB597: 16.32 ± 1.05; 0 PDTC: 16.15 ± 1.16 0 Hydralazine: 15.72 ± 1.42). During and after repetitive drug injections, URB597 and PDTC group rats showed significantly increased mechanical threshold values, compared to vehicle-injected rats (1 to 4 URB597: 20.47 ± 1.83, 21.19 ± 1.34, 21.93 ± 1.52, and 24.19 ± 1.56; 1 to 4 PDTC: 21.12 ± 1.68, 21.98 ± 1.48, 22.79 ± 1.42, and 22.66 ± 1.60; 1–4 vehicle: 16.29 ± 1.46, 15.05 ± 1.58, 13.96 ± 1.77, and 13.79 ± 1.42). Although, hydralazine also attenuated mechanical allodynia in CPIP model rats, its analgesic effects were reduced after discontinuing the drug (1 to 4 Hydralazine: 21.05 ± 1.41, 20.93 ± 1.42, 18.60 ± 1.39, and 18.35 ± 1.77).

### 3.2. Cellular Expression of Nav1.7 in DRGs

**Figure 2:**
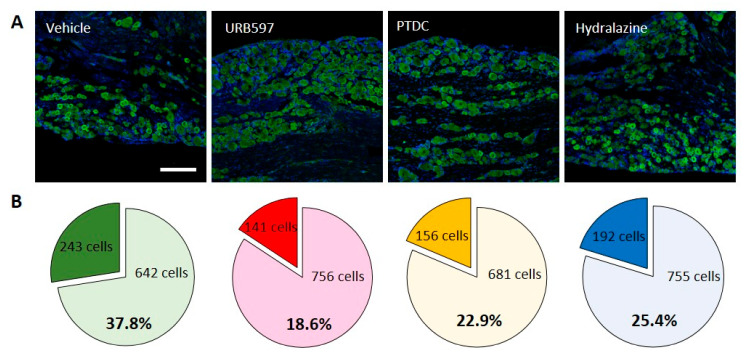
Activation of Nav1.7 channels in DRGs of the CPIP model. In DRG sections, immunohistochemical evidence showed that the expression of Nav1.7 increased in CPIP-injured rats. (**A**) Comparison of Nav1.7 expression in vehicle, URB597, PTDC, and Hydralazine injection groups. (**B**) Pie charts showing the percentage of DRG neurons expressing Nav1.7 among all treated drugs. The upper number indicates the number of Nav1.7-expressing neuron cells, and the lower number indicates the non-expressing neuron cells.

To further investigate molecular changes underlining pain after CPIP, we first examined levels of Nav1.7 expression in rat DRG neurons to determine its localization relative to analgesic markers. As shown in Figure 2A, immune fluorescent images of Nav1.7 antibody staining revealed nuclear Nav1.7 co-localized with nociceptive neurons in DRGs. IHC was performed to determine the cellular localization of Nav1.7 in rat DRGs at the end of behavioral tests. Consistent with behavioral changes, representative IHC images of DRGs from vehicle-treated rats show that the expression of Nav1.7 increased following CPIP induction. However, the URB597-, PTDC-, and hydralazine-treated rats showed lower expression of Nav1.7 in small DRG neurons following repetitive treatment (Figure 2A).

Nav1.7-expressing cells out of all neuronal cells were counted and calculated. In the vehicle group, 243/642 (Nav1.7-positive/non-positive) cells were counted. Conversely, in the URB597 group, reduced Nav1.7-positive cells were counted, compared to the vehicle group (141/756 cells). Furthermore, a similarly decreased expression of Nav1.7 was observed in PDTC and hydralazine group rats (PDTC 156/681; Hydralazine 192/755). The percentages of Nav1.7-expressing cells among DRG neurons are shown in individual pie charts (Figure 2B). More than 30% of the neurons expressed Nav1.7-positive signals after CPIP, and the expression thereof were reduced after drug treatment. These results indicated that drug treatment could modulate CPIP-induced pain.

### 3.3. Spatial and Temporal Differences in Neural Responses after Electrical Stimulation

**Figure 3:**
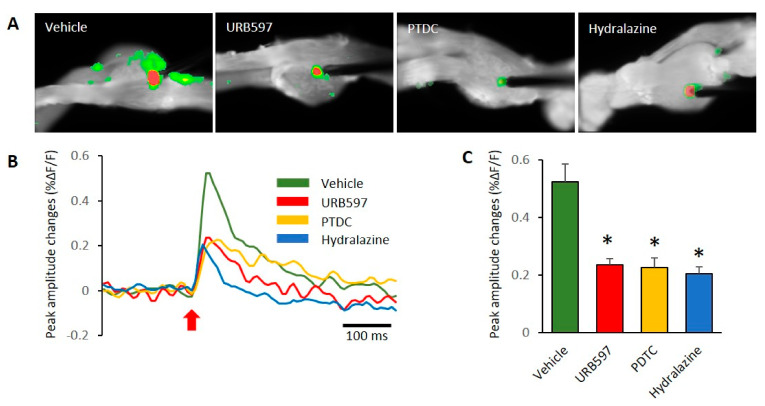
Comparison of DRG responses with electric stimulation in each group. (**A**) Comparison of VSD signals in the vehicle, URB597, PTDC, and hydralazine injection groups. (**B**) The typical time course of optical signals from DRG neurons. Red arrow represents the stimulus onset. Fluorescence changes: upward deflections of the signal indicate depolarization after electrical stimulation. (**C**) The reduced signals were observed in DRGs of drug-treated rats. *n* = 5 rats/group; one-way ANOVA followed by Tukey post hoc test was used for statistical analysis; data represent mean ± SEM; * *p* < 0.05.

In this study, we used VSD imaging to record membrane potential changes in rat DRGs. To observe neuronal activity corresponding with electrical stimulation, we stimulated the center of DRGs and recorded the resultant DRG neuronal activity. This allowed us to examine the spatial and temporal properties of DRG responses by electrical stimulation. In DRGs from the vehicle-treated group, VSD imaging revealed subthreshold activity spread over large regions of the DRGs after stimulation (Figure 3A). Images showing patterns of activity after electric stimulation are shown in Figure 3A, and an example of the association for VSD signals is shown in Figure 3B. We found pronounced differences between the vehicle and other groups of DRGs. The prominent difference was that responses to electrical stimulation after 200 ms were high in the vehicle group, as can be seen in Figure 3B. We used the center of electrode regions to collect temporal signals of DRG activation after stimulation. In the comparison of peak amplitude changes, vehicle DRGs showed significantly increased activity, compared to other drug treated groups (vehicle: 0.52 ± 0.06, URB597: 0.23 ± 0.02, PDTC: 0.22 ± 0.03, and Hydralazine: 0.20 ± 0.02). These peak amplitude responses in DRGs are shown in Figure 3C.

### 3.4. Expression Changes in Il-1β, Il-6, and TNFα in DRGs after Drug Application

**Figure 4:**
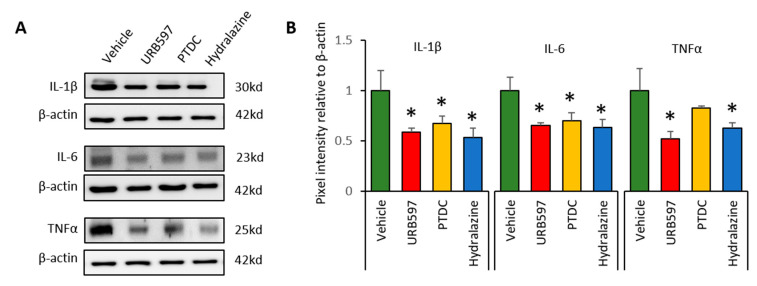
Changes in the relative density of IL-1β, IL-6 and TNFα protein in the ipsilateral DRGs (L3, L4 and L5). (**A**) Representative data indicate Western blotting for IL-1β, IL-6, and TNFα in DRGs. After repetitive injection of drugs, protein levels decreased. (**B**) Protein expression changes after drug application. *n* = 5 rats/group; one-way ANOVA followed by Tukey post hoc test was used for statistical analysis; data represent mean ± SEM; * *p* < 0.05.

To determine whether CPIP-induced inflammation reduced after drug application, we analyzed the expression of inflammatory cytokines in DRGs at the end of the experiments. Western blot analysis was conducted to detect the expression levels of IL-1β, IL-6, and TNFα. IL-1β, IL-6, and TNFα expression in the vehicle group was greater than that in the other groups (Figure 4A). Ratios of the expression of inflammatory factors are shown in Figure 4B. Compared to vehicle groups, IL-1β expression in the URB597, PDTC, and hydralazine groups was significantly reduced (vehicle: 1 ± 0.20, URB597: 0.58 ± 0.04, PDTC: 0.67 ± 0.07, and hydralazine: 0.53 ± 0.09). Similarly, expression of IL-6 and TNFα was also showed significantly lower in the drug-treated groups (IL-6, vehicle: 1 ± 0.13, URB597: 0.65 ± 0.02, PDTC: 0.70 ± 0.08, and hydralazine: 0.63 ± 0.07; TNFα, vehicle: 1 ± 0.21, URB597: 0.52 ± 0.07, PDTC: 0.82 ± 0.02, and hydralazine: 0.62 ± 0.05). These results suggested that drug application may be reduce inflammatory responses in DRGs from CPIP rats.

## 4. Discussion

In the present study, we evaluated the pain-relieving effects of URB597, PDTC, and hydralazine injections in an animal model of CRPS. To do so, Nav1.7 channel expression levels in DRGs were analyzed, and the analgesic effects after administration of the drugs were confirmed. We further investigated spatiotemporal neural activity changes in DRGs using VSD. Finally, we analyzed the expression of inflammatory factors in DRGs from CRPS rats to confirm the effects of the drug against CRPS. Changes in the nociceptive system in the animal model and the pattern of distinct pain reduction effects by URB597, PDTC, and hydralazine suggested the possibility of a novel treatment target for CRPS.

URB597 is a relatively selective inhibitor of the FAAH enzyme [22]. FAAH is a promising target for modulating endocannabinoid and fatty acid ethanolamide signaling, which may have important therapeutic potential [23]. Previous studies have demonstrated a significant increase in both mRNA and protein expression levels of cannabinoid 1 receptor, transient receptor potential vanilloid 1, and N-acyl phosphatidylethanolamine phospholipase D (NAPE-PLD) in the DRGs of neuropathy rats during the development or maintenance of pain [24,25]. Current studies indicate that the FAAH inhibitor URB597 reduces the nociceptive response caused by inflammatory and neuropathic pain in CRPS via systemic or intrathecal administration and that these pain-relieving effects can be blocked by cannabinoid receptor antagonists [26,27]. Such robust expression levels provide evidence that neuropathic pain is modulated through changes in FAAH signaling. Several studies have shown that FAAH inhibition attenuates mechanical and thermal hyperalgesia in CRPS models [26,28,29]. In addition, NAPE-PLD, which is synthesized in tissue, elevates anandamide levels. This activity may be interpreted as an endogenous defense mechanism against pain. In this study, we observed significant increases in mechanical threshold values after injection of the URB597 in CRPS rats. In addition, we performed immunohistochemistry and optical imaging study to examine neuronal excitation induced by CRPS and observed inhibitory effects for URB597 on pain signals. Distinct changes in neuronal activation under CRPS conditions after URB597 treatment indicated that URB597 could alleviate CRPS-induced pain responses. Although there are limited studies on the cannabinoid signaling pathway in CRPS, URB597 modulation has been shown to be involved in processing pain signals.

Nuclear factor-κB (NF-κB), a transcription factor of DNA, is involved in cellular responses to various stresses, such as cytokines, free radicals, heavy metals, and bacterial or viral antigens [30]. NF-κB is also known to be involved in various inflammatory diseases and to mediate cytokine expression [31]. Overexpression or inappropriate activation of NF-κB has been implicated in a number of pathological mechanisms of disease stemming from inflammation [32]. In the present study, PDTC, a selective antioxidant and inhibitor of NF-κB, was used to reduce inflammatory and pain responses after CRPS in rats. Previous studies have indicated that PDTC treatment inhibits superoxide anion-induced NF-κB activation, cytokine production, and oxidative stress in the paw and spinal cord of rats [30,33,34]. Furthermore, intrathecal administration of PDTC has been used to successfully inhibit superoxide anion-induced mechanical hyperalgesia, thermal hyperalgesia, and inflammatory responses in peripheral regions [33]. In our study, we observed behavioral and cellular changes after the induction of CRPS, and allodynia was relieved by administration of the NF-κB inhibitor PDTC. Additionally, reduced expression of inflammatory factors after PDTC treatment indicated that PDTC could modulate the pain-related inflammatory response in CRPS. However, we were unable to show a direct relationship between PDTC administration and decreased NF-κB activity. Although reduced NF-κB activation was not examined in this study, our findings may at least provide new clues of use to future research into the treat mechanisms of poorly understood CRPS.

Hydralazine, which is used to treat high blood pressure, is in a class of medications called vasodilators. The drug works by relaxing the blood vessels so that blood can flow more easily through the body. In this study, hydralazine was used for the purposes of recovering I-R injury after CPIP and reducing subsequent pain responses. Although hydralazine has been reported to be problematic in patients with a history of acute aortic dissection, stroke, coronary artery disease, or heart failure [35], our study showed that it can effectively reduce pain after CRPS. Hydralazine may have prevented microvascular damage after CPIP through inhibition of NO production by the iNOS/NO pathway and inhibition of oxidative stress, inflammatory response, and cell death by mitochondrial dependent pathways [36]. Our results confirmed that the pain modulation effects were reduced when discontinuing injections of the drug and indicated that the vasodilation effect controlled by hydralazine in CRPS is less effective than its effects on pain after URB597 and PDTC treatment.

## 5. Conclusions

We demonstrated the pain modulation effects of URB597, PDTC, and hydralazine on CRPS in rats. Each drug inhibited mechanical allodynia, expression of Nav1.7 channels, stimulus-evoked neuronal activation, and the release of inflammatory factors in DRGs. Currently no viable treatment strategies are available for CRPS due to an incomplete understanding of the underlying mechanisms of CRPS triggered by multiple causes [37,38]. Although numerous therapeutic strategies have been proposed and tested, none has fully met clinical demand. Our results may provide rationale for the use URB597, PDTC, and hydralazine as new treatment options with which to moderate pain in CRPS.

## Data Availability

Not applicable.
